# Cost-Effectiveness of Male Circumcision for HIV Prevention in a South African Setting 

**DOI:** 10.1371/journal.pmed.0030517

**Published:** 2006-12-26

**Authors:** James G Kahn, Elliot Marseille, Bertran Auvert

**Affiliations:** 1 Institute for Health Policy Studies, University of California San Francisco, San Francisco, California, United States of America; 2 Health Strategies International, Orinda, California, United States of America; 3 Institut National de la Santé et de la Recherche Médicale (INSERM), U687, Saint Maurice, France; 4 University of Versailles-Saint Quentin, Faculté de Médecine Paris-Ile-de-France-Ouest, Saint Maurice, France; 5 Hôpital Ambroise Pare (AP-HP), Boulogne, France; Massachusetts General Hospital and Harvard Medical School, United States of America

## Abstract

**Background:**

Consistent with observational studies, a randomized controlled intervention trial of adult male circumcision (MC) conducted in the general population in Orange Farm (OF) (Gauteng Province, South Africa) demonstrated a protective effect against HIV acquisition of 60%. The objective of this study is to present the first cost-effectiveness analysis of the use of MC as an intervention to reduce the spread of HIV in sub-Saharan Africa.

**Methods and Findings:**

Cost-effectiveness was modeled for 1,000 MCs done within a general adult male population. Intervention costs included performing MC and treatment of adverse events. HIV prevalence was estimated from published estimates and incidence among susceptible subjects calculated assuming a steady-state epidemic. Effectiveness was defined as the number of HIV infections averted (HIA), which was estimated by dynamically projecting over 20 years the reduction in HIV incidence observed in the OF trial, including secondary transmission to women. Net savings were calculated with adjustment for the averted lifetime duration cost of HIV treatment. Sensitivity analyses examined the effects of input uncertainty and program coverage. All results were discounted to the present at 3% per year.

For Gauteng Province, assuming full coverage of the MC intervention, with a 2005 adult male prevalence of 25.6%, 1,000 circumcisions would avert an estimated 308 (80% CI 189–428) infections over 20 years. The cost is $181 (80% CI $117–$306) per HIA, and net savings are $2.4 million (80% CI $1.3 million to $3.6 million). Cost-effectiveness is sensitive to the costs of MC and of averted HIV treatment, the protective effect of MC, and HIV prevalence. With an HIV prevalence of 8.4%, the cost per HIA is $551 (80% CI $344–$1,071) and net savings are $753,000 (80% CI $0.3 million to $1.2 million). Cost-effectiveness improves by less than 10% when MC intervention coverage is 50% of full coverage.

**Conclusions:**

In settings in sub-Saharan Africa with high or moderate HIV prevalence among the general population, adult MC is likely to be a cost-effective HIV prevention strategy, even when it has a low coverage. MC generates large net savings after adjustment for averted HIV medical costs.

## Abstract 2

Based on data from a trial of adult male circumcision to reduce the spread of HIV, a modeling study shows this intervention to be a cost-effective strategy.

## Editors' Summary

**Background.:**

Preventing the spread of HIV is an enormous challenge of great importance worldwide. In 2005, HIV/AIDS was responsible for around 3 million deaths, of which approximately one-third were in sub-Saharan Africa. HIV is spread from one person to another in three main ways: through unprotected sex; through contaminated blood or blood products (for example when shared needles are used); and from mother to child (during pregnancy, labor, and breastfeeding). Many strategies for preventing HIV focus on reducing risky behaviors. For example, condoms used correctly are effective at preventing HIV infection, and many countries now aim to promote condom use, together with other approaches that will reduce the risk of getting HIV. However, it is unlikely that strategies involving large-scale changes in behavior will ever be completely effective. Recently, much attention has focused on the possibility that circumcision might provide men with some protection against getting HIV. The results of a trial carried out in South Africa, the ANRS 1265 trial (published in PLoS Medicine in October 2005) seem to support this theory, and additional trials are being carried out in Kenya and Uganda. The results from these further trials will help determine whether, and to what extent, the effect of circumcision seen in the South African trial is true more generally.

**Why Was This Study Done?:**

The investigators who had carried out the South African circumcision trial wanted to find out how the economic aspects of this prevention strategy would compare with other strategies for prevention of HIV. Specifically, they wanted to know how much male circumcision would cost overall, per HIV infection prevented, as compared with the cost of other strategies. They also wanted to understand whether circumcision would be “cost-saving.” In other words, would the cost of performing the operation (together with the cost of treating any adverse effects suffered by the men who were circumcised) be offset by the costs of treatment for HIV infections that the intervention prevented? Getting this information is crucial before health policy makers can decide what strategies for preventing HIV are most appropriate for their country.

**What Did the Researchers Do and Find?:**

In this study, the researchers carried out a set of mathematical calculations, using the results from the ANRS 1265 trial, together with some other data and background assumptions. Their model was based on a hypothetical group of 1,000 men, all of whom would be circumcised. The researchers calculated that in such a hypothetical group, the cost of providing male circumcision, per HIV infection prevented, would be around $180. Overall, this procedure seemed to be cost-saving when the cost of HIV treatment was also factored in; around $2.4 million would be saved for the 1,000 men circumcised.

**What Do These Findings Mean?:**

These results suggest that, assuming the results of the South African trial are generally true, male circumcision would reduce the cost of health care in South Africa, mainly through savings on the cost of HIV treatment. The overall cost of male circumcision, per HIV infection prevented, is reasonable as compared to the costs of other strategies for prevention of HIV. There would also be implications for HIV prevention programs in other African countries. However, these estimates are based on the data from one trial only. The World Health Organization does not currently recommend the promotion of male circumcision for prevention of HIV. Meanwhile, proven strategies for preventing HIV exist, and more information is available from the links below.

**Additional Information.:**

Please access these Web sites via the online version of this summary at http://dx.doi.org/10.1371/journal.pmed.0030517.

- The World Health Organization has an HIV/AIDS program site providing comprehensive information on the HIV/AIDS epidemic worldwide
- General information and resources from the US Centers for Disease Control and Prevention on preventing HIV/AIDS
- Fact sheet from the Joint United Nations Programme on HIV/AIDS about male circumcision and HIV
- Results of the ANRS 1265 Trial evaluating male circumcision for HIV prevention were published in *PLoS Medicine* in October 2005; two related “Perspective” articles were also published in the same issue by Nandi Siegfried and Peter Cleaton-Jones

## Introduction

Despite hopeful signs of abatement in a few countries, the HIV epidemic continues to spread [1]. In the face of this challenge, substantial new resources have been committed to expand access to both prevention and treatment. These include the United States' Emergency Plan for AIDS Relief, the World Bank's Multi Country AIDS Projects, the World Health Organization's 3 by 5 Initiative, and the Global Fund to Fight AIDS, Tuberculosis and Malaria [2–4]. As the financial and political commitment to prevention intensifies, so too does the sense of urgency to identify new and effective methods to reduce transmission. The geographical area with the highest numbers of infected persons is sub-Saharan Africa [5]. Success in this setting remains critical to stemming the global epidemic.

Most prevention strategies among adults in sub-Saharan Africa focus on altering key HIV risk behaviors and treatment of sexually transmitted infections (STIs), but often lack confirmatory evidence of reduced HIV incidence. The most effective interventions, such as sex worker programs, apply only to limited groups [6,7]. Effective vaccines remain elusive and are unlikely to be ready for many years [8,9]. Thus, new biologically based interventions that provide partial but durable protection not dependent on sustained behavioral change could make a significant contribution.

Observational studies have repeatedly shown that male circumcision (MC) offers substantial protection. A meta-analysis of 27 observation studies of MC in sub-Saharan Africa found that 21 showed evidence of reduced risk of infection (crude relative risk [RR] = 0.52; 95% confidence interval [CI] 0.40–0.68). In the subset of 15 studies that adjusted for potential confounding variables, circumcision appeared to reduce risk by over half (adjusted RR = 0.42; 95% CI 0.34–0.54) [10]. Ecological studies have shown that countries where males are circumcised, and generally at an early age, are less affected than others by the HIV epidemic. These studies argue for the likelihood of a durable protective effect [11]. The protective effect of MC is biologically plausible [12].

Recently, the first randomized clinical trial of MC, conducted in Orange Farm (OF), South Africa, provided the first experimental evidence. This trial found a risk reduction similar to that in meta-analyses (RR = 0.40; CI 0.24–0.68) [13]. Two additional randomized, controlled trials (RCTs) are underway, in Kenya and Uganda, and will be completed by 2007.

In addition to high efficacy, MC is a one-time medical intervention with partial but likely durable effect, potentially applicable to all uncircumcised men. In populations with null or low existing circumcision rates, the scope of public health benefit is potentially high [14]. MC in adults and children may be a key part of a broadened program of HIV prevention in the next years. There is already some adult male circumcision in this region; a 2002 Human Sciences Research Council study found that 35% of South African adults and young males have been circumcised, at a mean age of 15 years [15].

While funds for prevention have greatly increased in recent years, they still fall far short of what is needed [16–18]. It is thus appropriate to consider how MC compares with other prevention and treatment strategies in developing countries in terms of economic criteria. Recent reviews of HIV prevention cost-effectiveness suggest a range of $10 to more than $10,000 per HIV infection averted (HIA) [19,20].

South African studies on the cost-effectiveness of HIV interventions focus on mother-to-child transmission prevention interventions (with results ranging from cost saving if adjusted for averted medical care cost to $2,492 per HIA) [21–24] and the cost-effectiveness of ongoing antiretroviral therapy [25–27]. Provision of the female condom to sex workers was found to be cost saving if adjusted for averted medical care costs [28]. A study of rescreening for HIV during late pregnancy found net savings [29], and another study found that targeted STI treatment in sex workers costs $78 per disability-adjusted life year (DALY) [30]. Other studies did not use the outcome metric of costs per HIA or per life year saved [21,29–32]. No studies have been carried out on the cost-effectiveness of MC, a requirement before wide adoption should be undertaken [33].

The purpose of this analysis is (1) to assess the cost-effectiveness of MC for Gauteng Province, South Africa, where the randomized controlled trial took place, allowing comparison with other prevention strategies, and (2) using the analytic framework thus established, to estimate cost-effectiveness in sub-Saharan African settings with slightly different epidemiology or cost structure.

## Methods

### Overview

We estimated cost-effectiveness for a hypothetical cohort of 1,000 newly circumcised South African adult men in the general population. Base case values for inputs derived from the RCT in OF and, when not measured in the OF trial, from other studies or estimates [13]. Costs included performing the circumcisions, providing community publicity, and treating adverse events. Effectiveness, defined as number of HIA among adults, was calculated by projecting the reduction in HIV incidence observed in the RCT over 20 years, adjusted for epidemic effects that would alter benefits. Cost-effectiveness was calculated with and without adjustment for the averted lifetime cost of HIV treatment, with explicit assumptions about use of antiretroviral treatment (ART). We estimated the change in DALYs, a measure of the burden of disease. We performed sensitivity analyses for individual inputs, program coverage, and different geographic settings in sub-Saharan Africa.

### Model

We developed a deterministic cost and epidemiologic model using a computer spreadsheet (available on request from the authors). This model calculates the program cost of the intervention as the sum of the number of MC performed times the unit cost of MC, an estimated cost for publicity, and the frequency of adverse events times their unit costs. The adjusted cost subtracts the savings in medical costs due to averted HIV infections. The expected benefit of MC (HIV infections prevented) is calculated on the basis of the estimated HIV incidence in susceptible men and the reduction in that incidence due to MC. A linked epidemic model calculates indirect protection of women through lower HIV prevalence in men. The model assumes no direct benefit to the female partners of circumcised men since limited observational data suggest little or no protection of these partners [34,35]. The model also does not account for the impact on children that the indirect benefit to females would have.

We calculated the unadjusted program cost per HIA, for comparability with prior analyses. Since adjusted costs (i.e., reflecting averted HIV medical costs) were negative, we report net savings for the cohort. We also estimated the net reduction in DALYs accounting for HIV infections prevented and adverse events. We did not calculate the reference cost-effectiveness ratio (i.e., using a quality adjusted measure of life years in the denominator); with a negative numerator, this ratio is difficult to interpret and counter-intuitive in its response to changes in numerator or denominator [36]. Detail on key model features is provided below and in the Protocol S1.

Specific cost inputs are the observed cost (including publicity) per MC during the OF RCT, the cost of adverse events, and the lifetime cost of treating HIV/AIDS cases. Specific epidemiologic parameters include the proportion of susceptible persons (HIV-negative) in the population, HIV incidence rate, protective benefit of MC for males, increased risk due to risk compensation, and adjustment of HIV infections prevented due to epidemic dynamics. Input estimates were derived from the OF trial data and assigned ranges based on CIs established by the trial. For inputs not derivable from this trial, low and high values were chosen to encompass likely ranges.

The analysis adopts the perspective of a government health care payer in South Africa. Costs are for 2006, and future costs and benefits are discounted to 2006 at 3% annually, the rate recommended by the Panel on Cost-Effectiveness in Health and Medicine of the US Public Health Service and other analysts [36,37].

We conducted sensitivity analyses to assess the effect of uncertainty in input values. We used one-way sensitivity analyses for all inputs and report them for the six inputs that had the largest effects on results or for which the small effects were important to document. We used three-way sensitivity analysis to explore the combined effect of varying MC cost, protective effect, and an “epidemic multiplier” (described in “Effectiveness,” below). We conducted threshold analyses for these two inputs for $0 net cost and for risk compensation (i.e., increased risky sexual behavior) for stable HIV incidence (no HIA). We also performed multivariate Monte Carlo simulations, which estimate the aggregate uncertainty from all inputs (Crystal Ball, version 7.2, Decisioneering [http://www.decisioneering.com]). In a 100,000-trial simulation, all model inputs were varied simultaneously. Input parameters were assigned values using symmetrical beta distributions with the base case as the mean and the tails bounded by the ranges shown in Table 1. This allowed us to determine 95% and 80% CIs. (For simplicity, the interval between the tenth and 90th, and 2.5th and 97.5th, percentiles are referred to as the 80% or 95% CIs, respectively.) We repeated the base case Monte Carlo with a symmetrical uniform distribution.

**Table 1:**
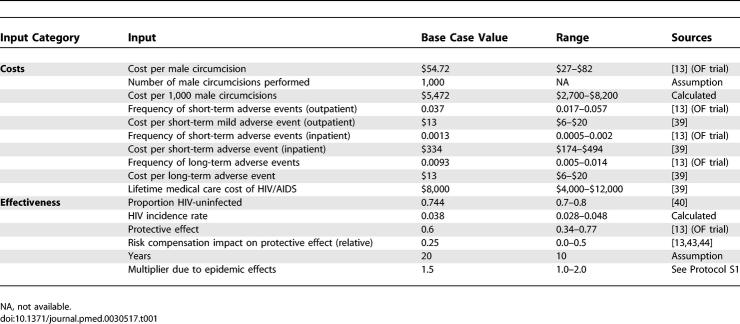
Input Values and Cost-Effectiveness Analysis of Male Circumcision

Finally, we examined three scenarios representing different epidemic settings, client characteristics, and coverage or uptake (i.e., number of MC, divided by the number of previously uncircumcised sexually active men in the community).

### Client Population

This analysis assumes a cohort of 1,000 adult males (older than 18 years) in the general population. This group is older than the relatively young subjects in the OF trial (aged 18–24) and thus has a higher HIV prevalence (see Discussion). In the base case we assume full MC program coverage. Other client scenarios examined include lower HIV prevalence and incidence, a focus on younger men (either initially or after circumcising all adult men), and lower MC program coverage.

### Costs

We considered all direct program and medical costs. The cost of performing an MC during the OF RCT was $47 (350 Rand, exchange rate 7.44 mid-2003), which was the average price charged by general practitioners in Gauteng Province for MC performed in their offices. This fee presumably includes all costs reasonably associated with providing the procedure (i.e., staff salaries, supplies, space, other practice costs). Training was not required due to familiarity with MC; two short meetings were used to standardize the procedure, and we assume no training cost. This cost is consistent with values reported from MC studies in Kenya that range from $13 to $77 [38]. We inflated the cost from mid-2003 to early 2006 using the US consumer price index, yielding a base case value of $49.72. To allow for community publicity, we added $5. (Input values are reported in Table 1.)

Our cost structure assumes zero additional training and physical infrastructure development costs in connection with high levels of circumcision coverage and does not assume that any economies of scale will be realized. We varied the cost per MC by ±50% to reflect these potential efficiencies or inefficiencies of scale-up.

In the OF trial, no death was attributable to MC, and the frequency of adverse events during surgery and within one month postoperatively was 3.8% (60/1,568). These included pain, excessive bleeding, infection, damage to the penis, swelling or hematoma, anesthesia-related events, excessive skin removed, insufficient skin removed, delayed wound healing, problems with urinating, and problems with appearance. Of these 60 adverse events, 58 were cured by a supplementary visit to a doctor and two necessitated a short hospitalization (2 d). The cost of the doctor visit including medication was estimated as $13.04, and the cost per hospital day at $167.20 [39]. These estimates are based on resources required for services to HIV-infected patients assuming 71% are served at a district-level hospital and 29% in a tertiary care facility, and thus are likely to overestimate the costs required for MC patients. At the end of follow-up, there were 11/1,185 (0.9%) adverse outcomes (problems with urinating, dissatisfaction with the appearance of the penis, mild or moderate erectile dysfunction, and torsion of penis). None of them led to permanent damage but they necessitated an outpatient visit to a doctor. Thus, the overall cost of adverse events standardized for 1,000 individuals was: 1,000 × ([58/1,568 × 13.04] + [2/1,568 × 2 × 167.2] + [0.9% ×13.04]) = $1,030.

The adverse events in the OF trial were immediately reported by general practitioners and were also reported by the participants and collated by a nurse at follow-up visits months after circumcision. Since this recall-based reporting may lead to underestimates of the rate of adverse events, we conducted a sensitivity analysis using three times this rate. These data may also overestimate adverse events attributable to MC (i.e., they are unadjusted for events in controls); however, we conservatively do not examine a reduced rate.

The lifetime cost of HIV treatment is based on a recent study in South Africa [39]. This study, which used data from pilot clinics for a prospective disease state model, estimated a lifetime discounted cost of $11,948 with ART and $3,793 without ART. We conservatively use $8,000 as the base case value, implying 50% access to ongoing ART, and explore a wide range from $4,000 to $12,000.

### Effectiveness

We define effectiveness as the number of HIV infections prevented per 1,000 newly circumcised men over a specified number of years (base case = 20). We calculate this effectiveness as the product of the number of HIV susceptibles, the HIV incidence rate, the protective effect of MC (adjusted for risk compensation), the projection period (in years), and an epidemic multiplier. This can be represented as: Effectiveness = number newly circumcised × (1 − HIV prevalence) × incidence rate × net protective effect × projection period × epidemic multiplier, where net protective effect = [1 − (1 − protective effect) × (1 + risk compensation)].

Each element of the equation is explained below.

The number newly circumcised is defined as the number of men in the cohort that will receive circumcision (set at 1,000). This includes men who are HIV-infected and uninfected.

The factor (1 − HIV prevalence) limits the analysis of effectiveness to those who are initially HIV-uninfected. The prevalence in adult men in Gauteng Province is 25.6% and we use this value [40]. Limiting the direct application of MC effectiveness to HIV acquisition among male susceptibles is conservative, but appropriately so given the HIV incidence outcome examined by the OF trial and the biological evidence. Those who are HIV-positive when circumcised do not contribute to effectiveness but do contribute to the cost.

HIV incidence rate represents the HIV acquisition risk that is lowered by MC. In a steady-state epidemic, an incidence of 0.038 is required to maintain an HIV prevalence of 25.6% (calculation available on request). For the base case analysis, we vary prevalence from 0.2 to 0.3 (corresponding to incidence of 2.8 per 100 person years and 4.8 per 100 person years, respectively). To examine results in a range of epidemic settings as well as circumcision for 18–24 year olds, we varied the prevalence down to 10% (incidence 1.4 per 100 person years).

The expression for net protective effect integrates the competing effects of biological protection by MC and potential risk compensation. The expression yields a net protection by combining the estimated RR of HIV acquisition due to each factor. If MC reduces the risk of acquisition by 60%, then the new RR of acquisition, absent behavior change, is 0.4 (i.e., 1.0–0.6). If risk compensation causes risk behavior to rise by 20%, this increases the RR of acquisition approximately linearly when incidence per time period is low [41], i.e., RR = 1.2. The combined effect of these two factors on RR is multiplicative, i.e., 0.4 × 1.2 = 0.48. Subtracting this RR from 1.0 yields the net protective effect (e.g., 0.52).

The protective effect found in the OF trial was 0.60, from a proportional hazards model. Statistically controlling for the increase in sexual risk behaviors in the intervention group had a minor impact on the estimated protective effect. We assume that the effect found in the trial is applicable to all men, and is causal without specifying a mechanism of action. We use 0.6 as the reduction in transmissibility, and separately model risk behavior increases due to risk compensation (defined below).

Risk compensation—i.e., increases in risk behavior precipitated by intervention-induced sense of reduced risk—is a major concern for any HIV-related prevention innovation [42]. A study in Kisumu (Kenya) showed that the sexual behavior of circumcised men was not different from that of uncircumcised men [43]. In contrast, a study in Uganda showed that circumcised men had a higher risk profile than uncircumcised men [44].

The MC RCT in OF found an 18% increase in the mean number of sexual contacts in intervention subjects versus controls. We assumed that risk compensation might be higher in a nonresearch program scale-up, and used a 25% increase in the frequency of risk behavior as the base case, varied in sensitivity analyses, from 0% to 50%. The 25% increase corresponds to a net protective effect of 0.50.

A projection period of 20 years was chosen for the base case analysis. This duration captures the persistent protective effect of male circumcision as well as delayed epidemic effects.

The epidemic multiplier was used to portray the effect of three factors that cause infections prevented to deviate from the simple product of HIA in the first year and the number of years projected. The first factor is that HIV infections prevented in men with circumcisions will lead to HIA in female partners in the community. In stable epidemics in a population with a negligible growth rate, each infected individual is responsible for an average of one HIV infection transmitted to others. Thus, if all other factors are constant, each infection prevented in HIV-negative men by MC (direct impact) might be expected on average to lead to one additional infection prevented in female partners. Because we consider a time period of 20 years in the base case, HIA late in that period (e.g., at year 15) would avert less than one infection.

A second factor that would enhance the benefits of MC is that, when a substantial number of men are circumcised and there is an indirect reduction in infections in women (as above), the lower HIV prevalence among women will further decrease risk to men. This will lead the epidemic to a new steady state with decreased prevalence and thus incidence among both women and men. The magnitude of this factor is best estimated with a time-dependent epidemic model. However, since it is one level of transmission further from the intervention (and thus delayed) and requires the slow decrease in HIV prevalence, we expect a somewhat smaller effect in the 20-year time frame.

Finally, the lower HIV prevalence in women resulting from the first infections averted by MC reduces the later incidence rate in susceptible men and hence the benefits of subsequent MC. This effect is also likely to be modest, as it operates through lower prevalence in women. It may vary with scale: an MC program with higher coverage yields a sharper drop in epidemic severity as compared with severity when the initial circumcisions occurred. We consider this factor as part of the overall epidemic multiplier and also separately.

In order to estimate the combined effect of these three factors, and to conduct sensitivity analyses, we adapted a simple dynamic epidemic modeling approach reported previously (described in Protocol S1). Overall, the “epidemic multiplier” representing all three factors was estimated as 1.53; we use 1.5 (range 1.0–2.0). While this simple model cannot capture the full range of epidemic dynamics as reported previously [14], it does permit a more accurate translation of protective effect in the RCT to epidemic benefit.

To compare effectiveness in preventing HIV to the health losses associated with adverse events, we calculated net DALYs. We estimated net DALYs by subtracting the increase in DALYs due to adverse events from the reduction in DALYs due to HIA. We calculated the reduction in DALYs for HIV by multiplying HIA by previously reported discounted DALY changes with ART (ten DALYs) and without (21 DALYs), assuming 50% on ART [45]. For increases in DALYs, we estimated the frequency and duration of adverse events from the OF RCT. We were explicitly pessimistic about the disutility of adverse events, assuming health state utility = 0 for most adverse events for their full duration. We used this approach because of the difficulty of obtaining utilities for these health states in this setting, and because even pessimistic assumptions yielded DALY increases from adverse events nearly two orders of magnitude smaller than DALY reductions from averted HIV infections. Detailed assumptions are available on request from JGK. We used the productivity weight for 25-year-olds (1.49) [46,47].

## Results

### Base Case Results

The cost of providing 1,000 MCs is estimated as $54,724 for the procedure and community publicity, and an additional $1,030 for management of adverse events. The total is $55,754. Base case results are presented in Table 2.

**Table 2:**
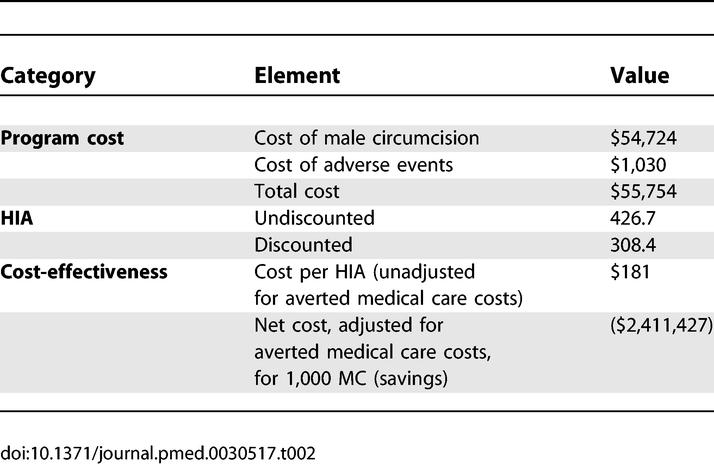
Program Cost, HIA, and Cost-Effectiveness of Male Circumcision

We estimate that over 20 years, the 1,000 circumcisions would avert 427 adult HIV infections (308 discounted to the present at 3%). This represents the combined effect of protection from the MC, 25% behavioral risk compensation, and a 1.5 epidemic multiplier reflecting secondary effects. An estimated two-thirds of infections are averted in men, and one-third in women.

We estimate that the HIA represent a reduction of 4.77 DALYs per MC (0.308 HIA per MC × 15.5 fewer DALYs per HIA). We pessimistically estimate a gain of 0.168 DALYs due to adverse events per MC, equal to 3.5% of DALYs averted. Thus, the net DALYs reduction per MC is 4.61, or 4,606 per 1,000 MCs.

The cost per HIA is estimated at $181 ($55,754/308). When adjusted for averted lifetime HIV medical costs of $8,000, the net savings for 1,000 MCs is $2,411,427 ($2,411 per MC).

### Sensitivity Analyses

The results below reflect variation in input values as reported in Table 1.

#### One-way sensitivity analyses.

**Figure 1:**
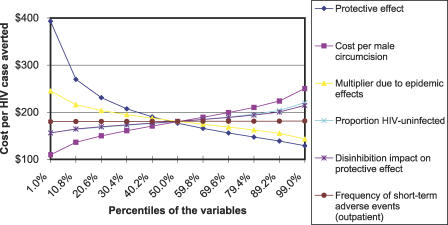
One-Way Sensitivity Analyses of the Cost per HIA Unadjusted for Anticipated Averted HIV Treatment Expenditures For each of the varied input values, results correspond to the ranges shown in Table 1. The 50th percentil corresponds with the base case. Similarly, the first and 99th percentiles approximate the low and high end of the ranges shown in Table 1, respectively. For example, the high-end cost per male circumcision is $82 (Table 1), which corresponds to the 99th percentile of the range, or $250 per HIA. This figure indicates that the unadjusted cost per HIA is most sensitive to uncertainty in the MC protective effect, cost per MC, and epidemic multiplier.

The cost per HIA, unadjusted for medical cost savings, is most sensitive to the estimated protective effect of MC, with values declining from $393 to $129 as protective effect increases from 0.34 to 0.77 (Figure 1). The cost of the MC procedure is the factor with the second largest effect on this outcome. Cost per case averted ranged from $110 to $250 as the cost of MC ranges from $27 to $82. Results are similarly sensitive to the multiplier due to epidemic effects, declining from $245 to $143 as the multiplier increases from 1.0 to 2.0. Results are relatively insensitive to variations in the value of other inputs, including HIV prevalence for this population (HIV prevalence values representing different populations are considered below under “Scenarios”). Results are extremely insensitive to the frequency of short-term adverse events, i.e., a 10-fold increase in the incidence of short-term adverse events decreases program net savings by 0.05%.

**Figure 2:**
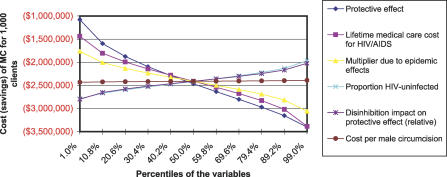
One-Way Sensitivity Analyses of the Cost of 1,000 Male Circumcisions after Deducting Averted HIV Treatment Costs For each of the varied input values, results shown correspond to the ranges shown in Table 1. The 50th percentile corresponds with the base case. Similarly, the first and 99th percentiles approximate the low and high end of the ranges shown in Table 1 respectively. Parentheses indicate savings. This figure indicates that the cost per HIA adjusted for averted HIV treatment costs is most sensitive to uncertainty in the MC protective effect, lifetime medical care cost for HIV/AIDS, and epidemic multiplier.

The factor with the greatest effect on overall costs adjusted for averted HIV medical expenditures was the protective effect of MC (Figure 2). Across the range of values we examined, 0.34–0.77, the net savings ranged from $1.1 million to $3.4 million. Other factors with large effects on results were the lifetime medical cost of HIV as a function of proportion on ART (savings of $1.4 million to $3.4 million) and the epidemic multiplier ($1.8 million to $3.1 million). An MC program breaks even or generates net costs only if the protective effect of MC is 0.21 or lower.

We examined the effect of a key method. If the time frame were ten years, HIA would drop to 155, with net savings of $1.18 million. A time frame of 50 years would increase HIA and net savings. We did not quantify these values due to limitations in our model's ability to account for population turnover, as well as large uncertainty regarding epidemic projections and prevention and treatment technologies.

#### Three-way sensitivity analysis.

Table 3 shows the effect on the cost per HIA when varying the protective effect of MC, its cost, and the epidemic multiplier. The least attractive outcome is obtained when the protective effect is near the low end of its range, 40%, program costs are at the high end, $100 per MC performed, and the epidemic multiplier is 1.0. In this pessimistic situation, the cost per HIA reaches $1,031, with net savings reduced to $716,000. At the other extreme, if the protective effect reached 70%, MC costs dropped to $30, and the epidemic multiplier was 2.0, the cost per HIA would be $70, and the net savings would reach $4.1 million.

**Table 3:**
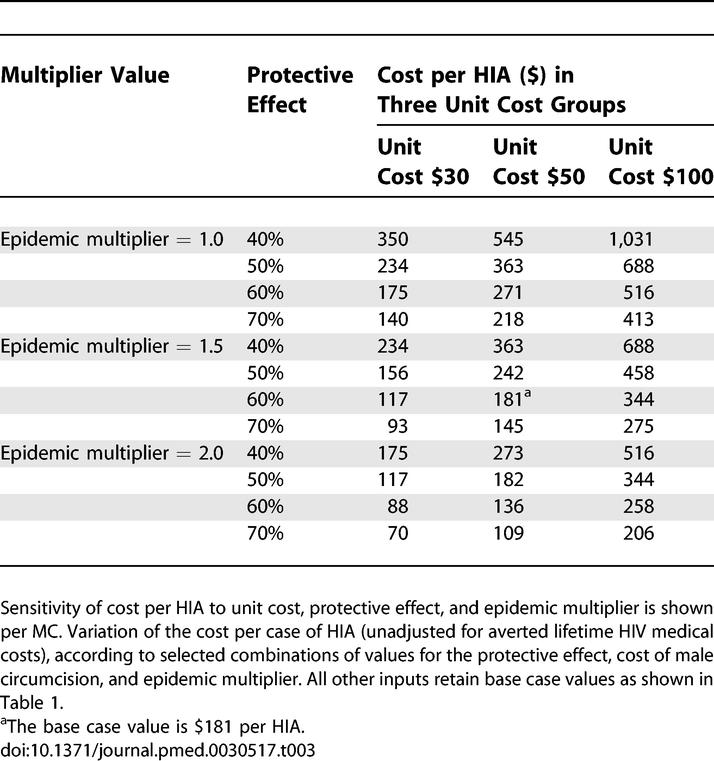
Three-Way Sensitivity Analysis

#### Threshold analyses.

In order for the MC program to have a net cost of zero for the base case epidemic setting, the protective effect would need to drop by two-thirds to 20.9% or the cost would need to increase 45-fold to $2,466.

In order for the MC to result in stable HIV incidence (no HIA), and using the best estimate of protective effect of 0.60, risk compensation would have to be 1.5 (i.e., 150% increase in risk behaviors). Assuming the worst case protective effect of 0.34, for stable HIV incidence the risk compensation would have to be 0.52 (as compared with a best estimate of 0.25).

#### Multivariate sensitivity analysis.

The multivariate Monte Carlo simulation showed that the number of discounted HIA over 20 years for 1,000 clients ranged from 130 to 486 at the 95% confidence level, and from 189 to 428 at the 80% confidence level. The unadjusted cost per case averted ranged from $95–$427 and from $117–$306 at the 95% and 80% confidence levels, respectively. Program cost adjusted for averted medical care costs ranged from savings of $0.9 million to $4.3 million at the 95% CI, and savings of $1.3 million to $3.6 million at the 80% confidence level.

When we repeated this simulation with uniform distributions for model input values, the resulting ranges in values of outcomes were wider but the finding of net savings remained. The number of discounted HIA was 99–574 (95% CI) and 151–475 (80% CI). The unadjusted cost per case averted was $71–$603 (95% CI) and $97–$390 (80% CI). Net savings ranged from $0.6 million to $5.4 million for the 95% and 80% confidence levels, respectively.

#### Scenario sensitivity analysis.

With an HIV prevalence of 8.4% and incidence of 0.01 per susceptible per year (i.e., a less severe steady state), the cost per HIA increases nearly 3-fold to $551 (80% CI $344–$1,071) and net savings are $753,000 (80% CI $330,000–$1.2 million). With prevalence still at 25.6% but incidence decreased to 0.01 per susceptible per year (i.e., a rapidly waning epidemic due to reduced risk behavior), there are 48 infections averted, at net savings of $264,246.

Performing MC in younger men (18–24 years old) may substantially improve cost-effectiveness, by averting the rise in prevalence that occurs with ongoing risk. With a starting HIV prevalence of 10% and incidence of 0.021 (as observed in the OF trial), our model predicts one-third more infections prevented over 20 years than with MC in all adult men. The cost per HIA would be $135.

If MC is performed in younger men after all adult men have been circumcised, cost-effectiveness becomes less favorable than the base case results. Assuming HIV prevalence and incidence of 8.5% and 0.015, respectively, our model predicts 20% fewer infections averted. The cost per infection averted would be $228.

The base case represents full coverage with MC. That is, all men eligible for circumcision receive it. If coverage were low (10%) despite recruitment at a cost of $5 per eligible, the cost would be $100 per MC performed. For 1,000 eligibles, there would be 100 MC, 33 discounted HIA, and net savings of $253,897.

The effect of coverage on effectiveness is small. When coverage (or uptake) is lowered by half as compared with the base case, the number of HIA per 1,000 MC increases by 1%. This effect reflects the balance of two forces. An MC program with high coverage yields a drop in epidemic severity, so that the residual HIV incidence affected by the last MC is substantially less than the HIV incidence affected by the first MC. However, specified drops in HIV incidence yield proportionally larger drops in prevalence in lower incidence ranges than in higher incidence ranges. The balance of these forces is the net scale effect. At different starting levels of HIV prevalence and incidence, we found a scale effect favoring smaller programs of 1%–10%.

## Discussion

Previous studies have demonstrated that MC reduces HIV transmission. The present analysis demonstrates that MC can lower health system costs. This is due to moderate implementation costs, high and durable protective effects, and the resulting averted HIV care costs. This finding is robust across a wide range of plausible parameter input values for South Africa, including lower effectiveness, higher costs, and lower HIV incidence.

This analysis also suggests that MC, at $181 in program cost per HIV infection prevented and cost saving when adjusted for averted medical costs, is amongst the most economically efficient of HIV prevention strategies in sub-Saharan Africa. The cost per HIA has been estimated at $68–$79 for peer education for sex workers, $58 for mass media, $10–$2,188 for condom distribution, $393–$482 for voluntary counseling and testing, $20–$2,198 for antiretroviral drugs to prevent mother-to-child transmission, $271–$514 for treatment of other sexually transmitted infection, and $7,288–$13,326 for school-based education. As noted below, the latter three interventions have mixed data on effectiveness, making the cost-effectiveness estimates less certain. MC is as economically favorable as inexpensive medical interventions for HIV, such as INH prophylaxis at $703 per fatal case averted or cost saving if averted secondary TB cases are included [19,20,48] and cotrimoxazole prophylaxis, which is also likely to be cost saving [49,50].

Other infectious disease interventions that are considered economically attractive in sub-Saharan Africa according to the World Bank's *Disease Control Priorities in Developing Countries* [51] have costs per averted DALY ranging from $2–$400. Standard childhood immunization costs $1–$5 per DALY. Malaria interventions including insecticide treated bed nets and residual household spraying cost $2–$24 per DALY. Improved quality and coverage of maternal and neonatal care are less cost-effective, $82–$409 per DALY [52]. We assume that these cost-effectiveness estimates were adjusted for medical care costs, suggesting net costs rather than net savings as with MC.

The evidence of the effectiveness of MC is consistent but not yet definitive. Results from the OF trial, consistent with a meta-analysis of observational studies, show a 60% protective effect. However, a limitation of this study is relying on only one clinical trial; two further trials are pending. Some other prevention interventions, though having an attractive cost per HIA in favorable circumstances, have often been found to lack evidence of effectiveness. This is true of mass media programs, school programs, and may also pertain to STI treatment [6,51].

Acceptability of MC remains a significant concern, due to strong cultural values regarding circumcision status and practices. Coercion should not be employed to overcome reluctance to obtain MC. Yet, high levels of acceptability of MC have been demonstrated in various African settings including Botswana, Kenya, Zimbabwe, and South Africa, where acceptability rates of 60%–70% were reported [53–56]. A community cross-sectional survey conducted in South Africa suggests that over 70% of noncircumcised men would elect circumcision if it protected against STIs. Two-thirds of the African population is already circumcised, including many African countries where all are circumcised, and where there is only a minority of Muslims (Benin, Cameroon, Democratic Republic of the Congo). Historical data suggest that MC can be increased (South Korea from 0% in 1900 to about 60% today) or decreased (Zulu were circumcised 200 years ago but not today) [57,58].

In some settings, low acceptability will reduce uptake. However, even if due to limited acceptability MC occupies a smaller HIV prevention niche, its high cost-effectiveness still argues for implementation of appropriately scaled programs. This may be especially true if acceptability evolves over time: “early adopters” pave the way for others later [59].

In the South African survey, 29% of circumcised and 22% of noncircumcised men believed that circumcision protects against HIV and other STIs [53]. A less encouraging result from this survey is that 30% and 18%, of circumcised and uncircumcised men respectively, believed that circumcision would permit them safely to have sex with multiple partners. Furthermore, circumcised men were more likely to report many lifetime partners than were their uncircumcised peers. These data underline the importance of further research regarding the educational campaigns and specific messages that would encourage participation while minimizing risk compensation.

These concerns also argue for capitalizing on the complementarities between MC and behaviorally based HIV prevention modalities such as condom promotion and counseling for partner reduction and other risk reduction. MC can serve as a portal for other male reproductive health services, including HIV prevention, which clients might otherwise not access. Even if not fully integrated with broader prevention services, MC facilities could routinely refer patients to programs that provide these services. For HIV-infected MC candidates this could also include referral for ART.

Complementarities are also present in the types of personnel required by MC programs. MC requires trained medical practitioners, but does not compete for the scarce supply of trained counselors, health educators, and field personnel who are the backbone of other HIV prevention and treatment modalities. Combined with the willingness in the OF RCT of general practitioners to perform MC at a reasonable price, these labor complementarities should enhance the feasibility of conducting an MC program without slowing other HIV activities. We are now planning research on the feasibility of scale-up.

Generalization of HIV prevention effectiveness and cost-effectiveness research is a universal concern. One issue is the effectiveness of prevention technology in other geographic settings, with different beliefs and behaviors. We believe that MC protective effect, based on biological rather than behavioral change, is more valid to generalize to other settings than are most HIV prevention strategies. For economic analyses, adjustment to local cost levels is necessary. Another issue is extrapolation from short-term trials to long-term effects. Again, we believe that the biological nature of MC fosters higher confidence in generalization. Risk compensation is an important phenomenon not reliably generalized from a short-term trial to other settings or time frames, and thus worthy of ongoing evaluation.

We believe that this analysis for South Africa applies to other sub-Saharan settings. The epidemic situation in South Africa (heterosexual spread, high HIV prevalence, low MC prevalence) is similar to most southern African countries (e.g., Lesotho, Zimbabwe, Swaziland, Botswana, Zambia). Although more men in the other sub-Saharan locations are circumcised, our economic findings are similar for large and incremental MC efforts. Our MC cost data are consistent with data from Kenya, and our sensitivity analyses confirm that MC is cost saving for a wide range of economic and epidemiologic conditions. We are therefore confident that our findings are relevant beyond South Africa.

We cannot be certain if trial-derived parameter values will differ from those found in actual practice. Some of those most subject to variability, such as risk compensation and the frequency of severe adverse events, have substantial influence on the cost-effectiveness results. High-risk compensation in the context of lower bound MC protective effect could even lead to the loss of HIV prevention benefit. However, the range of parameter values explored in the sensitivity analyses, including some that appear to be extremely pessimistic, provide substantial reassurance that MC can be effective and cost saving. Research on operating programs will permit refinement of key parameter values.

Unit costs may decline and cost-effectiveness rise following wide intervention adoption. This could arise from the usual economies attendant upon large volumes. In addition, it is possible that lower cost nonmedical doctor paramedics, nurses, and traditional circumcisers could be trained to perform MCs safely and successfully. It is also possible that benefits are greater than estimated here. This could occur, for example, if MC were found to confer protection on women in addition to the circumcised men. Our estimate did not take into account the prevention of HIV infection among newborns due to the indirect protective effect on adult women, which would also tend to lower cost-effectiveness.

By improving clinical outcomes or prevention, public health programs sometimes reduce future medical care costs. However, these medical care cost savings are often realized in a different budget. When the entity charged with funding the program does not realize the savings, it may be less motivated to implement a program of prevention than it would be if these savings accrued to its own budget. MC and HIV care are both in the medical budget rather than the public health budget. For this reason, decision makers considering implementation of an MC initiative would not only incur the costs of such a program but would also make savings in future HIV/AIDS care. On a cash-flow basis, the project should thus be attractive to the administering agency, yielding a stream of net savings starting approximately 6–8 years after implementation, when most MC clients would otherwise have started consuming medical care costs for HIV/AIDS treatment. This alignment of budgetary costs and benefits could thus raise the political and administrative acceptability of this proposal.

If adopted in the context of high-quality medical services and appropriate community and individually oriented health education programs, MC could contribute significantly to reducing HIV transmission in Southern Africa. Findings from this study suggest that MC could be highly cost-effective or could save health system funds. Botswana, Lesotho, Malawi, Mozambique, Namibia, South Africa, Swaziland, Uganda, Zambia, and Zimbabwe combine low MC prevalence with high HIV prevalence. These countries are therefore potentially high-priority candidates for implementation.

## Supporting Information

Protocol S1 Cost-Effectiveness of Male Circumcision for HIV Prevention in a South African Setting (32 KB DOC)

## Abbreviations

ART: antiretroviral therapy
CI: confidence interval
DALY: disability-adjusted life year
HIA: HIV infection(s) averted
MC: male circumcision
OF: Orange Farm
RCT: randomized controlled trial
RR: relative risk
STI: sexually transmitted infection

## References

[pmed-0030517-b001] UNAIDS (2004). 2004 report on the global HIV/AIDS epidemic: 4th global report.

[pmed-0030517-b002] US Department of State, Office of the US Global AIDS Coordinator (2004). The President's emergency plan for AIDS relief.

[pmed-0030517-b003] World Health Organization (2004). Scaling up antiretroviral therapy in resource-limited settings: Treatment guidelines for a public health approach: 2003 revision.

[pmed-0030517-b004] The Global Fund to fight AIDS, tuberculosis, and malaria (2005). A partnership to prevent and treat AIDS, tuberculosis, and malaria. Fact sheet, 21 January 2005.

[pmed-0030517-b005] UNAIDS (2005). AIDS Epidemic Update.

[pmed-0030517-b006] Marshall N, Marseille E, Bertozzi S, Dandona L, Kahn JG (2004). A systematic, quantitative review of existing data on HIV prevention effectiveness and the link between program outputs and risk reduction [poster]. XV International AIDS Conference.

[pmed-0030517-b007] Stover J, Bertozzi S, Gutierrez JP, Walker N, Stanecki KA (2006). The global impact of scaling-up HIV/AIDS prevention programs in low- and middle-income countries. Science.

[pmed-0030517-b008] Newman PA, Duan N, Rudy ET, Anton PA (2004). Challenges for HIV vaccine dissemination and clinical trial recruitment: If we build it, will they come?. AIDS Patient Care STDS.

[pmed-0030517-b009] Levy JA (2001). What can be achieved with an HIV vaccine?. Lancet.

[pmed-0030517-b010] Weiss HA, Quigley MA, Hayes RJ (2000). Male circumcision and risk of HIV infection in sub-Saharan Africa: A systematic review and meta-analysis. AIDS.

[pmed-0030517-b011] Moses S, Bradley JE, Nagelkerke NJ, Ronald AR, Ndinya-Achola JO (1990). Geographical patterns of male circumcision practices in Africa: Association with HIV seroprevalence. Int J Epidemiol.

[pmed-0030517-b012] Szabo R, Short RV (2000). How does male circumcision protect against HIV infection?. BMJ.

[pmed-0030517-b013] Auvert B, Taljaard D, Lagarde E, Sobngwi-Tambekou J, Sitta R (2005). Randomized, controlled intervention trial of male circumcision for reduction of HIV infection risk: The ANRS 1265 Trial. PLoS Med.

[pmed-0030517-b014] Williams BG, Lloyd-Smith JO, Gouws E, Hankins C, Getz WM (2006). The potential impact of male circumcision on HIV in Sub-Saharan Africa. PLoS Med.

[pmed-0030517-b015] Human Sciences Research Council (2002). Nelson Mandela/HSRC study of HIV/AIDS: South African national HIV prevalence, behavioural risks and mass media: Household survey 2002.

[pmed-0030517-b016] Schwartlander B, Stover J, Walker N, Bollinger L, Gutierrez JP (2001). Aids: Resource needs for HIV/AIDS. Science.

[pmed-0030517-b017] UNAIDS (2005). Resource needs for an expanded response to AIDS in low and middle income countries–“Making the money work”: The three “ones” in action. Discussion paper 9.

[pmed-0030517-b018] UNAIDS (2003). Progress report on the global response to the HIV/AIDS epidemic, 2003: Follow-up to the 2001 United Nations General Assembly Special Session on HIV/AIDS: Executive Summary.

[pmed-0030517-b019] Creese A, Floyd K, Alban A, Guinness L (2002). Cost-effectiveness of HIV/AIDS interventions in Africa: A systematic review of the evidence. Lancet.

[pmed-0030517-b020] Hogan DR, Baltussen R, Hayashi C, Lauer JA, Salomon JA (2005). Cost effectiveness analysis of strategies to combat HIV/AIDS in developing countries. BMJ.

[pmed-0030517-b021] Soderlund N, Zwi K, Kinghorn A, Gray G (1999). Prevention of vertical transmission of HIV: Analysis of cost effectiveness of options available in South Africa. BMJ.

[pmed-0030517-b022] Wilkinson D, Floyd K, Gilks CF (1998). Antiretroviral drugs as a public health intervention for pregnant HIV-infected women in rural South Africa: An issue of cost-effectiveness and capacity. AIDS.

[pmed-0030517-b023] Wilkinson D, Floyd K, Gilks CF (2000). National and provincial estimated costs and cost effectiveness of a programme to reduce mother-to-child HIV transmission in South Africa. S Afr Med J.

[pmed-0030517-b024] Skordis J, Nattrass N (2002). Paying to waste lives: The affordability of reducing mother-to-child transmission of HIV in South Africa. J Health Econ.

[pmed-0030517-b025] Boulle A, Kenyon C, Skordis J, Wood R (2002). Exploring the costs of a limited public sector antiretroviral treatment programme in South Africa. S Afr Med J.

[pmed-0030517-b026] Kenyon C, Skordis J, Boulle A, Pillay K (2003). The ART of rationing–the need for a new approach to rationing health interventions. S Afr Med J.

[pmed-0030517-b027] Cleary SBA, McIntyre D, Coetzee D (2004). Cost-effectiveness of antiretroviral treatment for HIV positive adults in a South African Township.

[pmed-0030517-b028] Marseille E, Kahn JG, Billinghurst K, Saba J (2001). Cost-effectiveness of the female condom in preventing HIV and STDs in commercial sex workers in rural South Africa. Soc Sci Med.

[pmed-0030517-b029] Wilkinson D, Floyd K, Gilks CF (2000). National and provincial estimated costs and cost effectiveness of a programme to reduce mother-to-child HIV transmission in South Africa. S Afr Med J.

[pmed-0030517-b030] Soorapanth S, Sansom S, Bulterys M, Besser M, Theron G (2006). Cost-effectiveness of HIV rescreening during late pregnancy to prevent mother-to-child HIV transmission in South Africa and other resource-limited settings. J Acquir Immune Defic Syndr.

[pmed-0030517-b031] Harrison A, Karim SA, Floyd K, Lombard C, Lurie M (2000). Syndrome packets and health worker training improve sexually transmitted disease case management in rural South Africa: Randomized controlled trial. AIDS.

[pmed-0030517-b032] Vickerman P, Terris-Prestholt F, Delany S, Kumaranayake L, Rees H (2006). Are targeted HIV prevention activities still cost-effective in high prevalence settings? Results from an STI treatment intervention for sex workers in Hillbrow, South Africa. Sex Transm Dis.

[pmed-0030517-b033] Atashili J (2006). Adult male circumcision to prevent HIV?. Int J Infect Dis.

[pmed-0030517-b034] Bonner K (2001). Male circumcision as an HIV control strategy: Not a “natural condom”. Reprod Health Matters.

[pmed-0030517-b035] Hunter DJ, Maggwa BN, Mati JK, Tukei PM, Mbugua S (1994). Sexual behavior, sexually transmitted diseases, male circumcision and risk of HIV infection among women in Nairobi, Kenya. AIDS.

[pmed-0030517-b036] Gold M, Siegel JE, Russell LB, Weinstein MC (1996). Cost-effectiveness in health and medicine.

[pmed-0030517-b037] Drummond MF, O'Brien B, Stoddart GL, Torrance GW (1997). Methods for the economic evaluation of health care programmes.

[pmed-0030517-b038] Krieger JN, Bailey RC, Opeya J, Ayieko B, Opiyo F (2005). Adult male circumcision: Results of a standardized procedure in Kisumu District, Kenya. BJU Int.

[pmed-0030517-b039] Cleary S, Boulle A, McIntyre D, Coetzee D (2004). Cost-effectiveness of antiretroviral treatment for HIV-positive adults in a South African township.

[pmed-0030517-b040] Dorrington RE, Bradshaw D, Budlender D (2002). HIV/AIDS profile of the provinces of South Africa—Indicators for 2002.

[pmed-0030517-b041] Pinkerton SD, Abramson PR (1996). Occasional condom use and HIV risk reduction. J Acquir Immune Defic Syndr Hum Retrovirol.

[pmed-0030517-b042] Cassell MM, Halperin DT, Shelton JD, Stanton D (2006). Risk compensation: The Achilles' heel of innovations in HIV prevention?. BMJ.

[pmed-0030517-b043] Auvert B, Buve A, Lagarde E, Kahindo M, Chege J (2001). Male circumcision and HIV infection in four cities in sub-Saharan Africa. AIDS 15 Suppl.

[pmed-0030517-b044] Bailey RC, Neema S, Othieno R (1999). Sexual behaviors and other HIV risk factors in circumcised and uncircumcised men in Uganda. J Acquir Immune Defic Syndr.

[pmed-0030517-b045] Marseille E, Saba J, Muyingo S, Kahn JG (2006). The costs and benefits of private sector provision of treatment to HIV-infected employees in Kampala, Uganda. AIDS.

[pmed-0030517-b046] Murray CJ, Lopez AD (1996). The global burden of disease: A comprehensive assessment of the mortality and disability from diseases, injuries, and risk factors in 1990 and projected to 2020.

[pmed-0030517-b047] Murray CJ (1994). Quantifying the burden of disease: The technical basis for disability-adjusted life years. Bull World Health Organ.

[pmed-0030517-b048] Bell JC, Rose DN, Sacks HS (1999). Tuberculosis preventive therapy for HIV-infected people in sub-Saharan Africa is cost-effective. AIDS.

[pmed-0030517-b049] Yazdanpanah Y, Losina E, Anglaret X, Goldie SJ, Walensky RP (2005). Clinical impact and cost-effectiveness of co-trimoxazole prophylaxis in patients with HIV/AIDS in Cote d'Ivoire: A trial-based analysis. AIDS.

[pmed-0030517-b050] Bachmann MO (2006). Effectiveness and cost effectiveness of early and late prevention of HIV/AIDS progression with antiretrovirals or antibiotics in Southern African adults. AIDS Care.

[pmed-0030517-b051] Korenromp EL, White RG, Orroth KK, Bakker R, Kamali A (2005). Determinants of the impact of sexually transmitted infection treatment on prevention of HIV infection: A synthesis of evidence from the Mwanza, Rakai, and Masaka intervention trials. J Infect Dis 191 Suppl.

[pmed-0030517-b052] Jamison DT, Breman JG, Measham AR, Alleyne G, Claeson M (2006). Disease control priorities in developing countries. 2nd edition.

[pmed-0030517-b053] Lagarde E, Dirk T, Puren A, Reathe RT, Bertran A (2003). Acceptability of male circumcision as a tool for preventing HIV infection in a highly infected community in South Africa. AIDS.

[pmed-0030517-b054] Kebaabetswe P, Lockman S, Mogwe S, Mandevu R, Thior I (2003). Male circumcision: An acceptable strategy for HIV prevention in Botswana. Sex Transm Infect.

[pmed-0030517-b055] Mattson CL, Bailey RC, Muga R, Poulussen R, Onyango T (2005). Acceptability of male circumcision and predictors of circumcision preference among men and women in Nyanza Province, Kenya. AIDS Care.

[pmed-0030517-b056] Halperin DT, Fritz K, McFarland W, Woelk G (2005). Acceptability of adult male circumcision for sexually transmitted disease and HIV prevention in Zimbabwe. Sex Transm Dis.

[pmed-0030517-b057] Kim DS, Lee JY, Pang MG (1999). Male circumcision: A South Korean perspective. BJU Int 83 Suppl.

[pmed-0030517-b058] Monteoro-Ferreira A (2005). Reevaluating Zulu religion: An Afro-centric analysis. J Black Stud.

[pmed-0030517-b059] Rogers E (1995). Diffusion of innovations.

