# Supplementary material for: Cost-Effectiveness of Male Circumcision for HIV Prevention in a South African Setting
Source: PLoS Med. 2006 Dec 26;3(12):e517. doi: 10.1371/journal.pmed.0030517 (PMC1716193; doi:10.1371/journal.pmed.0030517)
Supplement: Supplementary file 1 [file pmed.0030517.sd001.doc]

**Protocol S1**

**Cost-effectiveness of Male Circumcision for HIV prevention in a South African setting**

**Epidemic model**

To estimate an “epidemic multiplier” capturing the joint effect of three factors which enhance or suppress the protective effect of male circumcision (see text and below), and to conduct sensitivity analyses, we adapted a simple epidemic modeling approach reported previously. [1, 2] This approach portrays risk populations rather than individuals, and is based on basic principles of HIV epidemic dynamics such as the relationship of HIV prevalence to HIV incidence and exit rate. For an epidemic in steady state, HIV prevalence and incidence are in a ratio which represents the full set of risk factors (e.g., numbers of partners, frequency of sex, use of condoms, and risk of HIV acquisition per exposure) that translate exposure to HIV in partners (i.e., HIV prevalence) to new infections in susceptibles (i.e., incidence). This ratio may differ for men and women. In the absence of risk reduction, the prevalence and incidence (and their ratio) remain stable over time. With risk reduction, such as due to MC, the ratio of incidence to prevalence falls, in proportion to the magnitude of the risk reduction and coverage of the intervention. For MC, HIV incidence in men falls in proportion to the protective effect observed in the RCT, corrected for risk compensation. The model is dynamic, portraying 20 years in annual steps. Over time, prevalence in males decreases, followed by prevalence in females. Incidence also declines due to the lower prevalence. To estimate the epidemic multiplier, we compared the number of infections predicted for men and women with different reductions in risk.

We found that for every infection averted in men, an additional 0.48 infections were averted in women. We also found that the infections averted in men exceeded the risk reduction by 5% (e.g., with a 50% risk reduction, 52.5% of the infections were averted), representing the benefit to men of lower HIV prevalence in women. Finally, we found that the first half of male circumcisions resulted in a 1% reduction in the epidemic benefit from the last MCs, representing the suppression of benefit due to moderated epidemic environment. Overall, the epidemic modifier was estimated as 1.53.

**Demonstration that Protective Effect and Risk Compensation are Multiplicative**

Here we justify our simplifying assumption that protective effect and risk compensation interact multiplicatively in their effect on HIV incidence.

Specify that p is the probability of transmission of HIV from an HIV+ woman to a man per unprotected contact (or partnership), and h is the HIV prevalence among women. Then after n sexual contacts (or n partnerships), a HIV-negative man has a probability of becoming infected expressed as a Bernoulli formula, i =1-(1-ph)^n^. In other words, the likelihood of infection is the cumulative probability of not being infected in each contact (1-ph), over n contacts. If the transmissibility becomes xp (x = 1- protective effect of MC) and the number of sexual contacts becomes kn (k = increase of sexual activity due to risk compensation), then the probability of infection becomes i_mc_= 1-(1-xph)^kn^.

If nph<<1, then the expression 1-(1-ph)^n^ approximates the sum of individual risk episodes (i.e., the last risky episode confers nearly the same risk as the first), and the Bernoulli formulation can be approximated by i = nph. Similarly, if knph<<1 (or less strictly, knxph << 1), then i_mc_= 1-(1-xph)^kn^ can be approximated by i_mc_= knxph. In this expression, reduced risk due to MC (x) interacts multiplicatively with increase in sexual activity (k) in determining the new level of incidence. For example, if x = 0.4 and k = 1.25, and no other factors change, then the ratio of knxph to nph is determined by 0.4 * 1.25 = 0.5. A similar linear effect has been demonstrated for condom use [3].

Finally, for our time step of one year, HIV incidence (inferred from prevalence) = 0.038 without MC, which meets the test of being << 1. This incidence is consistent with the estimate transmission risk per contact (p) of approximately 1 per 500, HIV prevalence in females of 20%, and 100 unprotected annual sex episodes. With MC, transmission probability drops 60% in our base case and therefore even with significant risk compensation incidence remains << 1.

**Citations:**

1.	Kahn JG, Kegeles SM, Hays R, Beltzer N. (2001) Cost-effectiveness of the Mpowerment Project, a community-level intervention for young gay men. J Acquir Immune Defic Syndr. 27:482-491.

2.	Kahn JG. (1996) The cost-effectiveness of HIV prevention targeting: how much more bang for the buck? Am J Public Health. 86:1709-1712.

3.	Pinkerton SD, Abramson PR. (1996) Occasional condom use and HIV risk reduction. J Acquir Immune Defic Syndr. 13:456-460.
